# A Knowledge Transfer Method from Multi-Channel to Single-Channel EEG Toward Real-World Emotion Recognition Deployment

**DOI:** 10.3390/bioengineering13101223

**Published:** 2026-10-19

**Authors:** Yanru Gu, Hanzhen Ouyang, Zihua Ouyang, Lishengsa Yue, Huajuan Mao, Weihui Dai, Yan Kang

**Affiliations:** 1 School of Management, Fudan University, Shanghai 200433, China; guyanru@fudan.edu.cn (Y.G.); ouyanghanzhen@fudan.edu.cn (H.O.); 23307100151@m.fudan.edu.cn (Z.O.); 2 Teaching and Research Department, Shanghai Institute of Science and Technology Management, Shanghai 201800, China; 3 College of Transportation, Tongji University, Shanghai 201804, China; 2014yuelishengsa@tongji.edu.cn; 4 The First Affiliated Hospital of Naval Medical University, Shanghai 200433, China; 5 School of Humanities and Management Sciences, Southwest Medical University, Luzhou 646000, China

**Keywords:** EEG, emotion recognition, knowledge transfer, single-channel EEG, knowledge distillation

## Abstract

Multi-channel electroencephalography (EEG) provides spatial information for emotion recognition but requires more complex signal acquisition than single-channel EEG. This study proposes a multi-channel-to-single-channel knowledge transfer framework that uses multi-channel recordings during training and one selected physical EEG channel at inference. A 32-channel teacher is pretrained, and Sparsemax-based gating is jointly optimized with a student using classification, logits distillation, frequency-band consistency, and gating regularization losses. The frequency-band objective compares signal-level spectral statistics of the multi-channel recordings and the gated surrogate input. After channel locking, the student is retrained on the selected physical signal. Binary Arousal and Valence classification was evaluated on DEAP using 32-fold leave-one-subject-out cross-validation. The proposed achieved higher subject-averaged balanced accuracy and F1-score than Channel-matched CE in both tasks, although component effects varied across tasks and metrics. These findings support the evaluated training approach under single-channel input constraints and provide a methodological basis for simplifying EEG acquisition.

## 1. Introduction

Emotion state recognition is an important research topic in affective computing, brain–computer interfaces (BCIs), wearable health monitoring, and human–machine interaction. Compared with external behavioral signals such as facial expressions, speech, and physical behaviors, electroencephalography (EEG) signals can provide continuous and dynamic physiological information at the neural activity level, and thus have been widely used for emotion state recognition [1,2]. However, most existing EEG-based emotion recognition studies rely on multi-channel EEG acquisition. Multi-channel systems can exploit spatial distribution information across different brain regions and usually achieve promising recognition performance under laboratory conditions. Nevertheless, in real-world scenarios, multi-channel EEG devices often suffer from complicated wearing procedures, long preparation time, heavy hardware burden, insufficient comfort, and potential interference with natural behaviors [3]. In applications requiring long-term or minimally intrusive monitoring, including mobile brain–computer interfaces, wearable health monitoring, psychological state assessment, and human–machine interaction, complex multi-electrode configurations increase deployment and maintenance costs, thereby limiting the practical usability of EEG-based emotion recognition systems. Therefore, real-world EEG emotion recognition requires not only improved recognition accuracy but also simplified hardware configuration, convenient wearing, and low-interference signal acquisition.

Reducing the number of EEG channels provides a direct solution to alleviate these deployment challenges. Existing EEG emotion recognition studies have explored lightweight EEG data configurations from the perspective of optimal channel selection, aiming to reduce data volume, computational complexity, and hardware requirements [4]. Meanwhile, studies on mobile, wireless, and ear-centered EEG systems have demonstrated that compact, low-cost, and unobtrusive acquisition configurations are more suitable for real-world applications [5,6,7]. Following this trend of channel reduction, single-channel EEG represents the most extreme and lightweight deployment configuration, where only one EEG channel is required. It should be noted that single-channel EEG is not a lossless replacement for multi-channel EEG; instead, it represents a practical trade-off that sacrifices partial spatial information in exchange for enhanced deployment feasibility. Therefore, the fundamental value of single-channel EEG emotion recognition lies in determining whether sufficient emotion-discriminative information can be preserved under the minimum channel configuration.

Despite its deployment advantages, single-channel EEG emotion recognition remains challenging. Compared with multi-channel EEG, a single channel provides only a local view of neural activity and cannot represent inter-channel functional connectivity. Studies on single-channel selection indicate that recognition performance is strongly dependent on the selected electrode, whereas connectivity features constructed from multiple channels can outperform features derived independently from individual channels [8,9]. Therefore, single-channel deployment involves a trade-off between hardware simplicity and the amount of spatial information available to the classifier. The key challenge is to preserve useful discriminative information learned from multi-channel EEG under an extremely limited input configuration.

To achieve single-channel EEG-based emotion recognition, existing studies have primarily investigated channel reduction and channel selection strategies. Traditional channel selection methods generally include filter-based and wrapper-based approaches. Filter-based methods rank EEG channels according to statistical measures such as correlation and mutual information. Although computationally efficient, these methods may ignore the interaction between channel selection and downstream classifiers [10,11]. Wrapper-based methods evaluate candidate channel subsets using classifier performance, which can improve task adaptation but require repeated model training and may increase computational cost and overfitting risk [11,12]. Recently, end-to-end learnable EEG channel selection methods have incorporated channel selection modules into deep neural networks and optimized discrete channel selection problems through differentiable relaxation techniques such as Gumbel-Softmax [13,14]. These studies provide important insights for single-channel EEG deployment, suggesting that channel selection can be integrated with model optimization rather than relying solely on manual expertise or offline searching. However, for real-world single-channel deployment, existing channel selection approaches still have two major limitations. First, learned channel weights or selected channel subsets do not necessarily correspond to a stable and deterministic EEG channel that can be directly used as the final single-channel input. Second, when channel search relies on soft weights, weighted combinations, or other surrogate inputs, a discrepancy may occur between the training stage and the deployment stage, where only the actual signal from one physical EEG channel is available.

Different from channel selection approaches, teacher–student knowledge transfer focuses on transferring discriminative knowledge learned by a multi-channel model to a lightweight model. Knowledge distillation transfers information from a high-capacity teacher to a smaller student model [15], and high-density-to-low-density distillation has been explored in EEG-based motor-imagery BCI [16]. Convolutional EEG decoders have demonstrated that temporal and spatial representations can be learned directly from raw EEG, including compact designs suitable for efficient implementation [17,18]. This strategy is particularly meaningful for real-world deployment scenarios: multi-channel EEG can provide comprehensive information during laboratory-based modeling or offline training, whereas practical applications require lightweight models operating with single-channel inputs. Conventional knowledge distillation mainly transfers class-discriminative knowledge by aligning the output distributions of teacher and student models, such as logits or soft targets. However, EEG-based emotion recognition relies not only on output-level classification information but also on neural rhythmic patterns distributed across different frequency bands. Previous studies have demonstrated that frequency bands and channel locations significantly influence EEG emotion recognition performance, with θ, α, β, and γ rhythms commonly used to characterize emotion-related neural activity patterns [19]. Therefore, in multi-to-single-channel knowledge transfer, output-level distillation alone may be insufficient to preserve the frequency-specific physiological information encoded by the multi-channel teacher model.

The aim of this study is to develop and evaluate a multi-channel-to-single-channel EEG knowledge transfer framework for emotion recognition, motivated by the need to simplify EEG acquisition. The framework uses multi-channel information during training, while requiring only one selected physical EEG channel at inference.

The main contributions of this study are summarized as follows:

(1) A Sparsemax-based channel-selection mechanism jointly learns channel weights and the student model, then uses a deterministic highest-weight rule to identify one physical EEG channel for inference.

(2) A frequency-band consistency objective supplements logits distillation by comparing the gated surrogate’s band log power with a channel-averaged reference from the multi-channel recordings.

(3) A channel-locked retraining strategy adapts the distilled student from the weighted surrogate to the selected physical signal using the classification loss.

The remainder of this paper is organized as follows. Section 2 reviews related studies on limited-channel EEG recognition, knowledge transfer, and preservation of frequency-level physiological information. Section 3 introduces the proposed sparse gating frequency-band distillation framework. Section 4 describes the experimental settings based on the DEAP dataset. Section 5 presents and analyzes the binary Arousal and Valence classification results. Section 6 discusses the findings and limitations, and Section 7 concludes the study.

## 2. Literature Review

### 2.1. Channel Reduction and Learnable Channel Selection for Limited-Channel EEG Emotion Recognition

Channel reduction and channel selection aim to decrease EEG acquisition and processing requirements while retaining emotion-discriminative information. Existing studies show that EEG features and electrode locations contribute differently to emotion recognition, providing a basis for identifying compact input configurations [4,20]. Recent studies have advanced neural-network applications in brain–computer interfaces, EEG-based state recognition, and affective computing [21,22,23]. Related work has also investigated graph-based relational modeling for conversational and multimodal emotion recognition [24,25] and cross-modal representation learning for multimodal sentiment analysis [26,27]. For EEG-based recognition, Ye et al. [28] proposed GraphEmotionNet, which combines adaptive graph learning and spatial–temporal attention to capture relationships among EEG channels for emotion classification. Its task-adaptive treatment of channel relationships offers a useful perspective for channel reduction: the retained input should be assessed in conjunction with the discriminative representations learned by the recognition model. This motivates coupling learnable channel weighting with downstream classification. From the perspective of methodological development, existing approaches can be broadly categorized into filter-based methods, wrapper-based methods, and end-to-end learnable channel selection methods. Filter-based methods typically rank EEG channels using task-independent statistical criteria, such as mutual information, and select channels with higher information content. Lan et al. proposed a mutual information maximization-based EEG channel selection method to identify more informative EEG channels [10]. These approaches offer high computational efficiency, relatively simple implementation, and independence from specific classifiers, making them suitable for preliminary channel screening. However, because selection is generally performed independently of the downstream classifier, filter criteria may fail to capture interactions between channel subsets and task-specific decision boundaries; statistically informative channels are not always the most discriminative for a particular model [11]. Therefore, for single-channel EEG deployment, filter-based methods can provide candidate channels but may not guarantee consistency between the selected channel and the optimization objective of deep learning models.

Wrapper-based methods evaluate different channel subsets according to the performance of target classifiers, allowing channel selection to directly serve specific recognition tasks. Marjit et al. [12] developed a hybrid sequential forward channel-selection method for EEG emotion recognition in which an SVM is used in the wrapper stage to evaluate candidate channel subsets. Compared with filter-based methods, wrapper-based methods can better reflect the relationship between channel selection and classification performance, thereby providing improved task adaptability. However, these methods usually require repeated model training or evaluation, resulting in high computational costs, particularly for high-dimensional EEG data and deep neural networks [11,12,13]. Furthermore, wrapper-based methods generally treat channel search as an independent process before or outside model training, making it difficult to integrate channel selection with representation learning and knowledge transfer in deep models.

Recently, end-to-end learnable channel selection methods have incorporated channel selection mechanisms into neural network training. Strypsteen and Bertrand developed a learnable EEG channel selection framework based on Gumbel-Softmax, enabling joint optimization of channel selection parameters and network parameters [13]. The significance of these studies lies in their ability to overcome the traditional pipeline of “channel selection followed by model training”, allowing channel selection to participate directly in model optimization and providing important methodological inspiration for single-channel EEG emotion recognition. Meanwhile, sparse probability mapping techniques offer new possibilities for more explicit and interpretable channel selection. Sparsemax can generate sparse probability distributions and is more suitable than conventional softmax for encouraging only a small number of candidates to receive non-zero weights [29]. This suggests that introducing sparsity into channel selection may enhance the concentration and interpretability of selected channels.

For single-channel inference, the selected input must correspond to one physical EEG channel rather than a weighted multi-channel combination. In differentiable selection, replacing the training-stage surrogate with that physical signal can introduce an input mismatch. Existing filter-based, wrapper-based, and learnable selection methods therefore motivate two connected design requirements: an explicit physical-channel choice and adaptation to the final single-channel input.

### 2.2. Knowledge Transfer and Preservation of Frequency-Level Physiological Information in EEG

Knowledge transfer provides a means of using information available during training to support models with restricted inputs. Knowledge distillation transfers supervisory information from a teacher to a student through output- or representation-level objectives [15,30]. In EEG-based motor-imagery recognition, similarity-keeping knowledge distillation has been used to guide low-density students using high-density recordings [16]. Recent brain–computer interface research also highlights the importance of matching transferred information to the target learning conditions. Chen et al. [31] developed a hybrid EEG–fNIRS framework that combines source-domain selection based on distributional similarity and validation performance with covariance and correlation alignment during transfer. This approach illustrates how task supervision can be complemented by constraints on the statistical relationships between source and target representations. For multi-channel-to-single-channel EEG learning, this provides a rationale for considering both class-discriminative supervision and the signal characteristics that can be retained under the student’s restricted input.

Conventional knowledge distillation mainly focuses on output-level class distributions and typically transfers the classification knowledge of teacher models through temperature-scaled soft targets or logits [15]. The advantage of such methods lies in their simplicity and broad applicability, enabling student models to learn the decision boundaries of teacher models. However, existing studies have also demonstrated that knowledge in teacher models is not limited to final outputs; intermediate representations can also provide valuable guidance for student model training. For example, FitNets introduced intermediate representations of teacher networks as “hints” to guide the training of thinner and deeper student networks, demonstrating that feature-level knowledge transfer can complement output-level distillation [32]. Related representation-level methods transfer attention maps or contrastive relationships between teacher and student features, further illustrating that effective distillation can operate beyond final logits [33,34]. This concept is particularly relevant to EEG emotion recognition: if only the final logits are aligned, the student model may learn the classification tendency of the teacher model but fail to capture intermediate representations or frequency-specific information related to EEG structures.

Frequency-specific information is an important consideration in EEG representation learning. Zheng and Lu [19] investigated the contributions of different frequency bands and electrode locations to emotion recognition, while Duan et al. [35] examined differential entropy and related spectral features for emotion classification. More recently, Chen et al. [22] constructed brain power maps from frequency-band power ratios and electrode positions for EEG-based pilot fatigue detection, integrating these structured inputs with convolutional, recurrent, and attention-based feature learning. This work illustrates how explicit spectral descriptors can be incorporated into deep EEG models. Together, these studies motivate examining frequency-band statistics as an auxiliary source of supervision during channel compression, alongside the class predictions supplied by a multi-channel teacher.

Adaptation to the final input configuration is another important consideration in knowledge transfer. Li et al. [36] combined residual feature learning with domain alignment to mitigate temporal covariate shift in emotion recognition from peripheral physiological signals. Their work highlights the importance of accounting for changes in the input distribution when reusing learned representations. In differentiable EEG channel selection, an input change occurs when a weighted multi-channel surrogate is replaced by the signal from one selected physical channel. The student must therefore learn to use the information available in that channel, providing a rationale for retraining on the selected signal after channel locking.

Taken together, task-adaptive channel learning, teacher-guided supervision, and explicit spectral modeling provide complementary foundations for limited-channel EEG recognition. These research directions motivate a framework that learns the retained physical channel while using multi-channel information to guide training, incorporates frequency-band statistics as an auxiliary constraint, and subsequently adapts the student to the selected single-channel input.

## 3. Model

### 3.1. Problem Definition

An EEG window sample is represented as:

(1) $$X=[x_{1},x_{2},\ldots ,x_{c}]\in R^{C\times T}$$

where *C* denotes the number of EEG channels, *T* represents the number of temporal sampling points within each EEG window, and *x_c_* ∈ *R^T^* denotes the time-series signal of the *c*-th EEG channel. In the experiments of this study, *C* = 32, and the input window length is set to 4 s. Given the sampling frequency *f_s_*, the number of sampling points in each EEG window is calculated as:

*T* = 4*f_s_* (2)

The corresponding label is defined as:

*y* ∈ {0,1} (3)

where *y* = 1 denotes High and *y* = 0 denotes Low for the task being evaluated.

For the binary classification task, the model outputs logits:

(4) $$z=[z_{0},z_{1}]\in R^{2}$$

The class probabilities are obtained through the softmax function:

(5) $$p_{k}=\frac{\exp(z_{k})}{\sum_{j=0}^{1}\exp(z_{j})},k\in \{0,1\}$$

The final predicted class is determined as:

(6) $$\hat{y}=\arg\max p_{k}$$

### 3.2. Proposed Framework 

**Figure 1:**
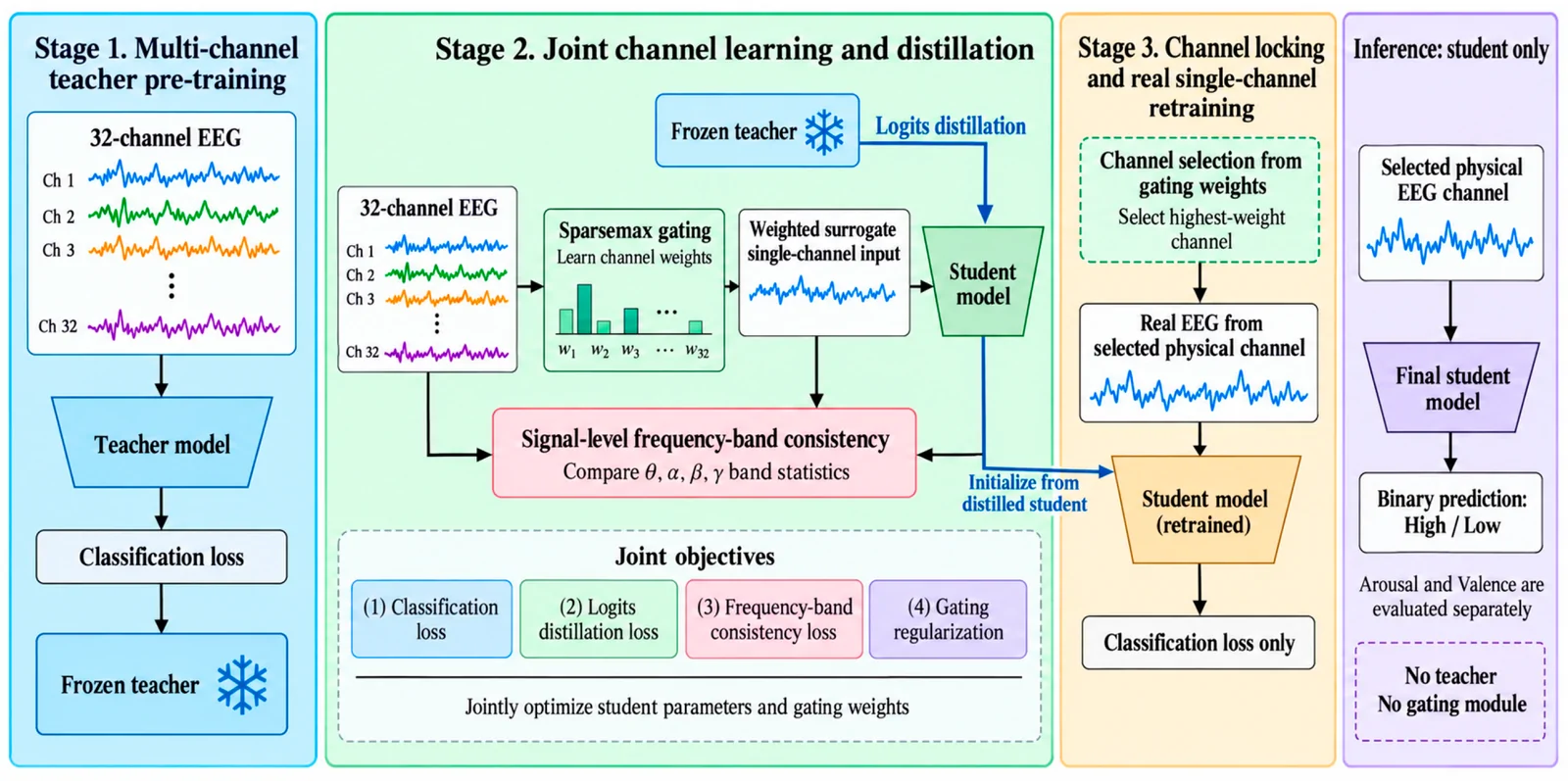
Overall Framework of the Proposed Method.

The overall framework of the proposed method is illustrated in Figure 1.

Step 1: Multi-channel teacher model pre-training. In this step, the teacher model is trained using the complete 32-channel EEG inputs to learn relatively comprehensive spatial distribution information and emotion-discriminative representations. After training, the teacher model is frozen and provides logit-level class-discriminative knowledge for subsequent training.

Step 2: Joint training with Sparsemax-based sparse gating distillation. This step represents the core component of the proposed framework. During training, the Sparsemax gating module learns channel weights and compresses multi-channel EEG signals into a single-channel surrogate input. The single-channel student model is trained using this surrogate input while simultaneously receiving supervision from four objectives: the hard-label classification loss, logits distillation loss, frequency-band consistency distillation loss, and sparse gating regularization constraint. During this stage, both the student model parameters and the gating weights are jointly optimized.

Step 3: Channel locking and real single-channel consistency retraining. After the distillation process, the EEG channel with the highest final gating weight is selected as the deployed single-channel input. The multi-channel weighted surrogate input is then removed, and the student model is further retrained using the actual single-channel EEG signal from the selected channel. The final model only requires one EEG channel as input during deployment.

### 3.3. Teacher Model Pre-Training

First, a multi-channel teacher model is trained using the complete multi-channel EEG input *X*:

(7) $$z_{T}=f_{T}(X;\theta_{T})$$

where *f_T_*(⋅) denotes the teacher model, *θ_T_* represents the parameters of the teacher model, and *z_T_* denotes the output logits of the teacher model. The teacher model adopts a lightweight temporal convolutional classifier as its backbone network. It is trained using the standard cross-entropy loss:

(8) $$L_{T}=L_{\mathrm{CE}}(y,z_{T})=-\sum_{k=0}^{1}1(y=k)\log p_{T,k}$$

where:

(9) $$p_{T}=\mathrm{softmax}(z_{T})$$

For binary classification samples, Equation (8) can be simplified as:

*L_T_* = −*y*log*p_T_*_,1_ − (1 − *y*)log*p_T_*_,0_ (10)

The purpose of the teacher model is to exploit the spatial distribution information contained in all *C*EEG channels and learn relatively comprehensive emotion-discriminative representations. After pre-training, the teacher model parameters *θ_T_* are frozen and remain unchanged in subsequent stages, serving only as the source of transferred knowledge. 

### 3.4. Joint Training with Sparse Gating Distillation

#### 3.4.1. Sparse Gating and Single-Channel Surrogate Input

During the distillation stage, a global sparse gating module is introduced to learn a channel compression strategy from multi-channel EEG signals, aiming to identify an EEG channel configuration suitable for single-channel input deployment. The learnable parameters of the gating module are defined as:

*a* = [*a*_1_, *a*_2_, …, *a_C_*] ∈ *R^C^* (11)

To convert the channel scores a into non-negative channel weights that sum to one, a Sparsemax mapping is applied in this study:

g = sparsemax(a)(12)

where:

*g* = [*g*_1_, *g*_2_, …, *g_C_*] ∈ *R^C^* (13)

subject to:

(14) $$g_{c}\geq 0,\;\sum_{c=1}^{C}g_{c}=1$$

*g_c_* = max(*a_c_* − *τ*(*a*),0) (15)

where *τ*(*a*) is an automatically determined threshold computed from *a*, ensuring that the resulting channel weights satisfy the probability simplex constraint (i.e., $\sum_{c=1}^{C}g_{c}=1$).

Compared with the conventional softmax function, Sparsemax produces a sparse probability distribution, allowing some channel weights to become exactly zero. This property makes it particularly suitable for channel selection tasks. Based on the learned gating weights *g*, the multi-channel EEG input is compressed into a single-channel surrogate input:

(16) $$x_{s}=\sum_{c=1}^{C}g_{c}x_{c}$$

where *X_s_* denotes the single-channel surrogate signal, which is constructed by linearly combining multiple EEG channels using the learned gating weights.

#### 3.4.2. Single-Channel Student Model

The student model takes this surrogate signal as input:

*z_S_* = *f_S_*(*x_s_*; *θ_S_*) (17)

where *f_S_*(⋅) denotes the student model, *θ_S_* represents the parameters of the student model, and *z_S_* denotes the output logits of the student model. The student model adopts the same type of lightweight temporal convolutional classifier as the teacher model, while taking a single-channel input.

#### 3.4.3. Logits Distillation

To transfer the class-discriminative knowledge learned by the multi-channel teacher model to the single-channel student model, this study first employs temperature-scaled logits distillation. Given the teacher model logits *z_T_* = *f_T_*(*X*) and the student model logits *z_S_* = *f_S_*(*X_S_*), let *τ* denote the temperature parameter. The softened output distributions of the teacher and student models are computed as:

(18) $$p_{T}^{\tau }=\mathrm{softmax}(\frac{z_{T}}{\tau })$$

(19) $$p_{S}^{\tau }=\mathrm{softmax}(\frac{z_{S}}{\tau })$$

When *τ* > 1, the softmax distribution becomes smoother, allowing the student model to learn not only the hard-label class predicted by the teacher model but also the teacher’s relative confidence across different classes.

The logits distillation loss is defined as:

(20) $$L_{\mathrm{KD}}=\tau^{2}\mathrm{KL}(p_{T}^{\tau }||p_{S}^{\tau })$$

where:

(21) $$\mathrm{KL}(p_{T}^{\tau }|\left|p_{S}^{\tau }\right)=\sum_{k=0}^{1}p_{T,k}^{\tau }\log\frac{p_{T,k}^{\tau }}{p_{S,k}^{\tau }}$$

This loss encourages the output distribution of the student model to align with that of the teacher model, thereby enabling the student model to acquire the class-discriminative knowledge learned by the teacher model.

#### 3.4.4. Frequency-Band Consistency Distillation

Aligning only the output logits may be insufficient to preserve the frequency-level physiological information that is essential for EEG-based emotion recognition. Therefore, this study further introduces a frequency-band consistency distillation objective to transfer frequency-level spectral representations from the multi-channel teacher model to the single-channel student model.

Let the set of frequency bands considered for EEG emotion recognition be defined as:

ℬ = {*θ*, *α*, *β*, *γ*} (22)

For the *c*-th channel *x_c_*(*t*), its frequency-domain representation is first obtained by the Fourier transform:

(23) $${\hat{x}}_{c}(f)=\sum_{t=1}^{T}x_{c}(t)e^{-j2\pi \mathrm{ft}/T}$$

The corresponding power spectrum can be expressed as:

(24) $$P_{c}(f)=\frac{1}{T}{|{\hat{x}}_{c}(f)|}^{2}$$

For a frequency band *b* ∈ *B*, let *F_b_* denote the corresponding set of frequencies. The average power of the *c*-th channel in frequency band *b* can be expressed as:

(25) $$E_{c}^{b}=\frac{1}{|F_{b}|}\sum_{f\in F_{b}}P_{c}(f)$$

To reduce the difference in power scale, the logarithmic power representation is adopted:

(26) $$r_{c}^{b}=\log(E_{c}^{b}+\varepsilon )$$

where *ε* is a small constant used to avoid taking the logarithm of zero.

For the teacher model, the multi-channel frequency-band features are obtained by channel aggregation:

(27) $$r_{T}^{b}=\frac{1}{C}\sum_{c=1}^{C}r_{c}^{b}$$

Therefore, the teacher-side frequency-band feature vector can be written as:

(28) $$r_{T}=[r_{T}^{\theta },r_{T}^{\alpha },r_{T}^{\beta },r_{T}^{\gamma }]$$

For the student model, the same spectral computation is performed on the single-channel surrogate input *x_s_*, yielding:

(29) $$P_{s}(f)=\frac{1}{T}{|{\hat{x}}_{s}(f)|}^{2}$$

The average power in frequency band *b* is:

(30) $$E_{s}^{b}=\frac{1}{|F_{b}|}\sum_{f\in F_{b}}P_{s}(f)$$

The logarithmic frequency-band feature is then obtained as:

(31) $$r_{s}^{b}=\log(E_{s}^{b}+\varepsilon )$$

The student-side frequency-band feature vector is:

(32) $$r_{S}=[r_{S}^{\theta },r_{S}^{\alpha },r_{S}^{\beta },r_{S}^{\gamma }]$$

The frequency-band consistency distillation loss is defined as:

(33) $$L_{\mathrm{band}}=\sum_{b\in B}{∥r_{T}^{b}-r_{S}^{b}∥}_{2}^{2}$$

This loss directly constrains the gated surrogate through the channel weights and affects student learning through its input. It does not attempt to fully recover the spatial information of multi-channel EEG; instead, it guides the student model to preserve part of the key rhythmic information under the single-channel setting.

#### 3.4.5. Joint Optimization Objective in the Distillation Stage

To further encourage the gating weights to converge toward a more explicit single-channel selection, a gating regularization term is introduced during the distillation stage. One possible form of the one-hot regularization is:

(34) $$L_{\mathrm{gate}}=1-∥g{∥}_{2}^{2}$$

When *g* follows a uniform distribution, $∥g{∥}_{2}^{2}$ is relatively small. As *g* approaches a one-hot distribution, $∥g{∥}_{2}^{2}$ approaches 1. Therefore, minimizing $1-∥g{∥}_{2}^{2}$ encourages the gating weights to concentrate on a single dominant channel.

During the sparse gating distillation stage, the student parameters and channel weights are jointly optimized using task classification, logits distillation, and signal-level frequency-band consistency losses. In addition, the gating regularization term is introduced to encourage sparsity in the channel weights. The joint loss function is defined as:

*L_distill_* = *L_CE_*(*y*,*f_S_*(*x_s_*)) + *λ_KD_L_KD_* + *λ_band_L_band_* + *λ_gate_L_gate_* (35)

where *λ_KD_*, *λ_band_*, and *λ_gate_* control the weights of the logits distillation loss, the frequency-band consistency distillation loss, and the gating regularization term, respectively.

The objective of this stage is to jointly optimize the student model parameters and the channel gating weights, enabling the student model to learn from the multi-channel teacher model under the single-channel surrogate input while gradually producing a more explicit channel selection result.

### 3.5. Consistency Retraining After Channel Locking

After the sparse gating distillation is completed, the final channel is selected according to the learned gating weights:

c* = arg max*g_c_* (36)

where c* denotes the selected EEG channel. Subsequently, the multi-channel weighted surrogate input is removed and replaced with the real single-channel signal from the selected channel: $x_{c^{*}}$.

During the consistency retraining stage, the student model is initialized with the parameters obtained from the distillation stage and is further trained using the real single-channel input $x_{c^{*}}$. At this stage, only the task classification loss is retained as the optimization objective:

(37) $$L_{\mathrm{retrain}}=L_{\mathrm{CE}}\left(y,f_{S}(x_{c^{*}})\right)$$

The purpose of this stage is to reduce the discrepancy between the surrogate input *x_s_* used during the distillation stage and the final real single-channel input $x_{c^{*}}$, thereby further adapting the student model to the single-channel signal used during deployment. Through retraining after channel locking, this study connects the differentiable channel search stage with the real single-channel input stage, thereby improving input consistency under the single-channel deployment constraint.

Algorithm 1 summarizes the three-stage training procedure and the final single-channel inference process of the proposed framework.
**Algorithm 1.** Multi-channel-to-single-channel EEG knowledge transfer**Input:** *D_tr_* and *D_val_*: windowed training and validation data for one task and LOSO fold; *f_T_* and *f_S_*: teacher and student models.**Settings:** *τ*, *λ_KD_*, *λ_band_*, *λ_gate_*(*e*); epoch budgets *E_T_*, *E_D_*, *E_R_*; learning rates *η_T_*, *η_D_*, *η_R_*.**Output:** $\mathrm{selected}\;\mathrm{physical}\;\mathrm{channel}\;c^{*}\;\mathrm{and}\;\mathrm{final}\;\mathrm{student}\;f_{S}(\cdot ;\;\theta_{S}^{*}).$**Notation:** *C* = 32; *Φ*(*x*) is the four-band log-power vector of signal *x* (Equations (23)–(32)); *B* = {*θ*, *α*, *β*, *γ*}.**Stage 1: Multi-channel teacher pre-training**Initialize teacher parameters *θ_T_*.for *e* = 1, …, *E_T_* doTrain *f_T_* for one epoch on *D_tr_* using Adam (*η_T_*) to minimize *L_CE_*(*y*, *f_T_*(*X*)).Save *θ_T_* when validation Balanced Accuracy improves.end forRestore the best teacher checkpoint and freeze *θ_T_*.**Stage 2: Joint training with Sparsemax-based sparse gating distillation**Initialize *θ_S_* and gate scores *a* so that sparsemax(*a*) is uniform.for *e* = 1, …, *E_D_* dofor each minibatch (*X*, *y*) in *D_tr_* do$g\;\leftarrow \;\mathrm{sparsemax}(a);x_{s}\;\leftarrow \;\sum_{c=1}^{C}g_{c}x_{c}$*z_T_* ← *f_T_*(*X*; *θ_T_*); *z_S_* ← *f_S_*(*x_s_*; *θ_S_*)$r_{T}\;\leftarrow \;(1/C)\sum_{c=1}^{C}\Phi (x_{c});r_{S}\;\leftarrow \;\Phi (x_{s})$*L_KD_* ← *τ*^2^ KL(softmax(*z_T_*/*τ*) ∥ softmax(*z_S_*/*τ*))$L_{\mathrm{band}}\;\leftarrow \;\sum_{b\in B}{\left(r_{T}^{b}-r_{S}^{b}\right)}^{2};L_{\mathrm{gate}}\;\leftarrow \;1-{\left\|g\right\|}_{2}^{2}$*L_distill_* ← *L_CE_*(*y*, *z_S_*) + *λ_KD_L_KD_* + *λ_band_L_band_* + *λ_gate_*(*e*)*L_gate_*Jointly update (*θ_S_*, *a*) using Adam (*η_D_*) to minimize *L_distill_*.end forSave (*θ_S_*, *a*) when validation Balanced Accuracy improves.end forRestore the best (*θ_S_*, *a*); set *g* ← sparsemax(*a*) and c* ← arg max*_c_ g_c_*.**Stage 3: Consistency retraining after channel locking**$\mathrm{Replace}\;\mathrm{the}\;\mathrm{surrogate}\;\mathrm{input}\;\mathrm{with}\;x_{c^{*}}$; remove gating and retain distilled *θ_S_*.for *e* = 1, …, *E_R_* do$\mathrm{Train}\;f_{S}\;\mathrm{for}\;\mathrm{one}\;\mathrm{epoch}\;\mathrm{on}\;D_{\mathrm{tr}}\;\mathrm{using}\;\mathrm{only}\;x_{c^{*}}$$,\;\mathrm{Adam}\;(\eta_{R}),\;\mathrm{and}\;L_{\mathrm{CE}}(y,\;f_{S}(x_{c^{*}})).$Save *θ_S_* when validation Balanced Accuracy on the locked channel improves.end for$\mathrm{Restore}\;\mathrm{the}\;\mathrm{best}\;\mathrm{retrained}\;\mathrm{parameters}\;\mathrm{as}\;\theta_{S}^{*}$$\;\mathrm{and}\;\mathrm{output}\;(c^{*},\;\theta_{S}^{*}).$**Inference: Single-channel student only; no teacher or gating module**$\mathrm{For}\;a\;4\;s\;\mathrm{window}\;u\;\mathrm{recorded}\;\mathrm{at}\;c^{*},\;\mathrm{compute}\;p\;\leftarrow \;\mathrm{softmax}(f_{S}(u;\;\theta_{S}^{*}))$ and *ŷ* ← arg max*_k_ p_k_*.For DEAP trial evaluation, average High-class probabilities over the 29 windows; classify as High when the mean is at least 0.5 and as Low otherwise.

## 4. Data

### 4.1. DEAP Dataset

This study employs the publicly available DEAP (Database for Emotion Analysis using Physiological Signals) dataset for evaluation [37]. The DEAP dataset contains multimodal physiological signals collected from 32 participants while they watched 40 music video stimuli. After viewing each video, each participant provided a subjective rating of his or her emotional state. In this study, Arousal and Valence are evaluated as separate binary classification tasks. For each task, ratings greater than or equal to 5 are labeled as High, whereas ratings below 5 are labeled as Low. In the subsequent evaluation, the High class is treated as the positive class and the Low class as the negative class. In this way, the original continuous ratings are converted into binary labels suitable for supervised learning.

For the EEG input, the first 32 EEG channels in the DEAP dataset are used as the multi-channel input. To be consistent with the proposed frequency-band consistency distillation objective, the EEG signals are processed within the frequency range of 4–45 Hz, with spectral features extracted from the θ, α, β, and γ frequency bands.

The first 3 s (384 samples) were excluded from each loaded 63 s trial, retaining the 60 s stimulus segment. Each segment was divided into 4 s windows with a 2 s step, yielding 29 windows per trial. All windows inherited their trial label. Windows served as training inputs, whereas original trials were the units for sample counting and evaluation. Trial-level predictions were obtained by averaging the High-class probabilities across the 29 windows and classifying a trial as High when the mean was at least 0.5.

After the above preprocessing, the original trial-level EEG data are converted into window-level EEG samples for teacher model pre-training, sparse gating distillation, and single-channel retraining after channel locking. The 32-channel teacher and all four single-channel methods used the same preprocessing, label definitions, and data partitions. They were evaluated using the same 32-fold leave-one-subject-out protocol for Arousal and Valence. In each fold, all trials and corresponding EEG windows from one participant were reserved exclusively for testing. Trials from the remaining participants were divided into training and validation subsets at an 80:20 ratio, with all windows from the same trial kept within the same subset. The training subset was used for parameter optimization, while the validation subset was used for model selection. The held-out participant was excluded from both processes and used only for testing. This procedure was repeated across all participants, with each participant serving as the test participant exactly once.

### 4.2. Baseline Methods

The following four single-channel methods are compared:

(1) Proposed: This is the complete method proposed in this study. It adopts a knowledge distillation framework from a multi-channel teacher model to a single-channel student model. A 32-channel teacher model is first trained, followed by learning a single-channel compression strategy through a Sparsemax-based global sparse gating module. During the distillation stage, Proposed jointly optimizes the task classification loss, the temperature-scaled logits distillation loss, the frequency-band consistency distillation loss over the θ, α, β, and γ bands, and the gating regularization term. Finally, after the target channel is determined, the student model is retrained using the real single-channel signal from the selected channel. This method is used to validate the effectiveness of the proposed framework for single-channel EEG emotion recognition.

(2) Logits-only: This is an ablation version of Proposed. It uses the same student network architecture and sparse gating strategy as Proposed, and is also guided by the multi-channel teacher model. However, the frequency-band consistency distillation loss is removed, leaving only the task classification loss and the temperature-scaled logits distillation loss. This setting is used to examine whether frequency-band consistency distillation can provide additional frequency-level information beyond output-level distillation, thereby improving the performance of the single-channel student model.

(3) Fixed: it is a fixed-channel single-channel baseline. This method uses neither knowledge distillation nor gating learning. Instead, an EEG channel is preselected as the input, and a single-channel classifier with the same architecture as the student model is trained. According to the experimental setting of this study, the Fixed method uses the Fp1 channel. This baseline is used to evaluate whether the learnable channel selection and distillation mechanisms provide advantages over training with a fixed single EEG channel.

(4) Channel-matched CE: it is introduced to control the influence of electrode location. In each LOSO fold, this baseline uses the same physical EEG channel selected by the proposed method as the input. It adopts the same student network architecture, preprocessing procedure, and data partitions as Proposed.

Unlike Proposed, Channel-matched CE is trained from random initialization using only the classification loss, without knowledge distillation, Sparsemax-based channel learning, or frequency-band consistency constraints. This baseline evaluates whether the proposed framework provides additional benefits beyond selecting a specific physical EEG channel.

Their comparison focuses on channel-selection and training strategies and the resulting recognition performance, rather than computational-efficiency differences arising from network size or inference implementation.

The default hyperparameter settings in the provided implementation are *T* = 4, *λ*_KD_ = 1.0, and *λ*_band_ = 0.5. In the recorded 10-epoch search runs, the gating regularization coefficient was zero during epochs 1–3 and then increased by 0.125 per epoch, reaching 0.875 at epoch 10. All stages use Adam, with default learning rates of 10^−3^ for teacher training, distillation, and Fixed baseline training, and 10^−4^ for channel-locked retraining. No learning-rate scheduler or automated hyperparameter search is implemented. The teacher and initial student networks use PyTorch’s (2.13.0) default layer initialization, while the gating weights are initially uniform. Channel-locked retraining starts from the distilled student parameters. No early stopping is used; each stage completes its prescribed training epochs and then restores the checkpoint with the highest validation Balanced Accuracy.

## 5. Results

### 5.1. Descriptive Statistical Analysis

Table 1 summarizes the trial-level label distributions for both tasks. Each LOSO fold contained 992 training trials, 248 validation trials, and 40 test trials. Across all 1280 distinct trials, Arousal included 965 Low trials (75.39%) and 315 High trials (24.61%), whereas Valence included 995 Low trials (77.73%) and 285 High trials (22.27%). Both tasks therefore exhibited class imbalance.

Because both tasks are imbalanced, predicting the majority class can yield relatively high accuracy. Balanced accuracy, F1-score, recall/TPR, TNR, and confusion matrices were therefore also used to assess minority-class recognition and false-positive control.

**Figure 2:**
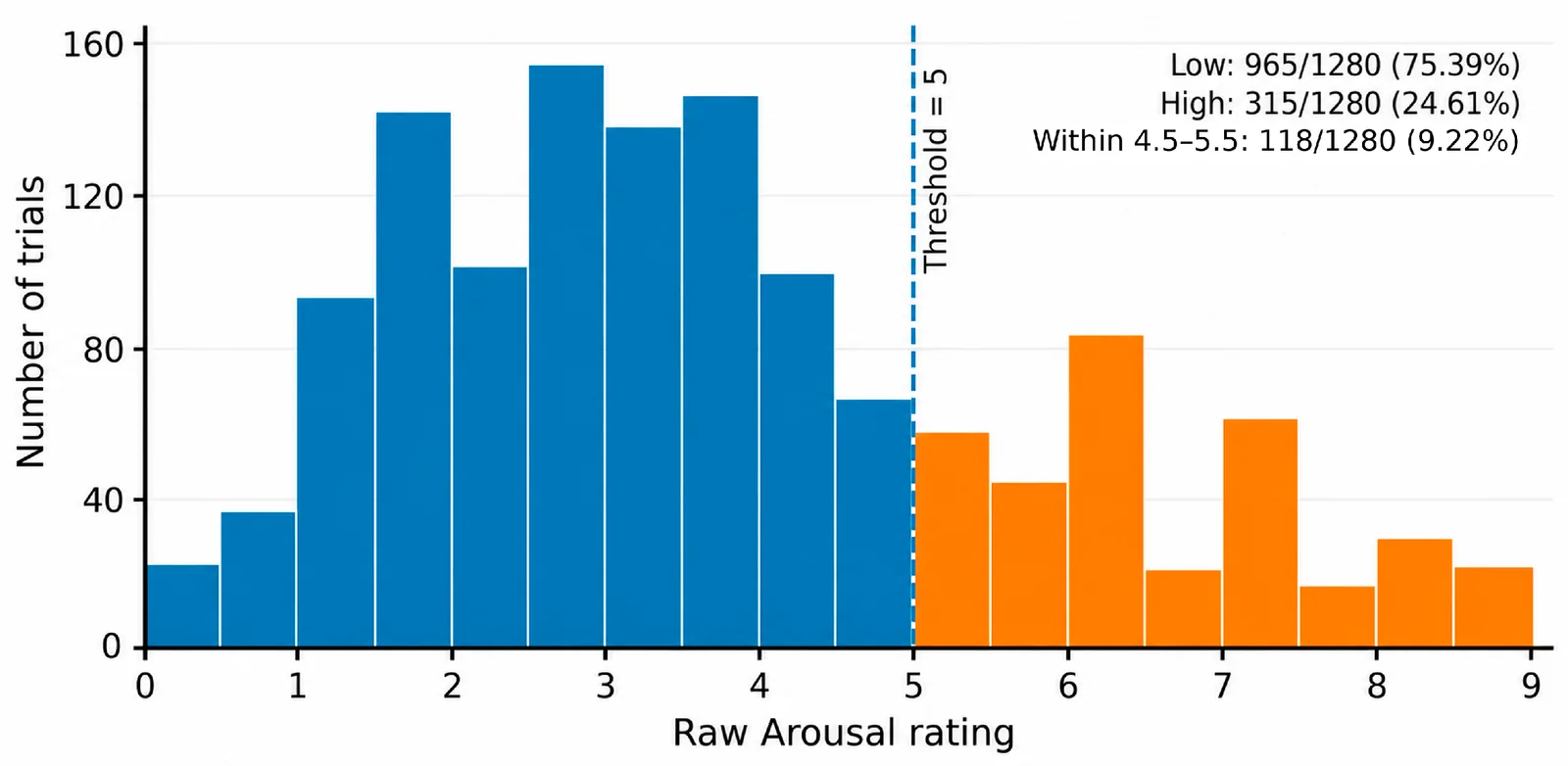
Arousal rating distribution and binarization threshold.

Figure 2 shows the Arousal rating distribution and the threshold of 5. The median was 3.315 and the third quartile was 4.990, placing the threshold toward the upper end of the distribution. Of the 1280 trials, 118 (9.22%) had ratings between 4.5 and 5.5, and 254 (19.84%) between 4.0 and 6.0. These concentrations near the threshold highlight the boundary imposed by converting continuous ratings into binary labels.

Table 2 reports all-channel mean log power for the θ, α, β, and γ bands, computed from 4 s windows and averaged at the trial level. The High–Low differences were 0.131, 0.070, 0.073, and 0.079 for Arousal, and −0.132, 0.003, −0.037, and −0.059 for Valence. Absolute Cohen’s d values were below 0.10 in both tasks, indicating small descriptive differences. These statistics do not establish that band power alone is sufficient for classification; the frequency-band objective is used as an auxiliary constraint.

### 5.2. Results on the Arousal and Valence Dimensions

**Figure 3:**
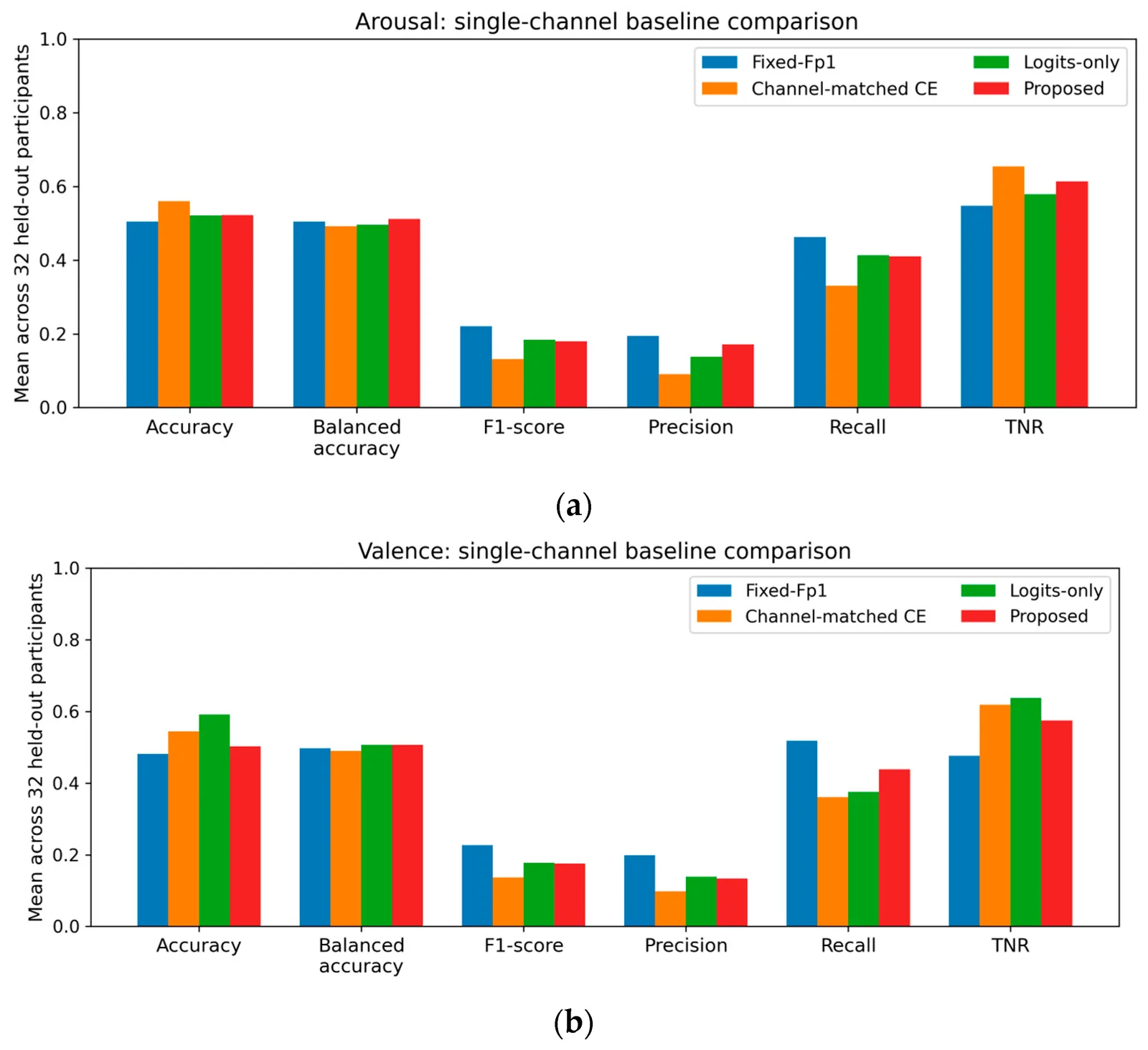
Performance of four single-channel methods: (**a**) Arousal and (**b**) Valence.

Figure 3 compares the four single-channel methods using trial-level metrics. Proposed and Channel-matched CE use the same physical channel in each fold, allowing their training strategies to be compared under matched electrode locations.

For Arousal, Proposed achieved higher subject-averaged balanced accuracy (0.5118 vs. 0.4923) and F1-score (0.1799 vs. 0.1315) than Channel-matched CE. For Valence, the corresponding values were 0.5063 vs. 0.4899 and 0.1756 vs. 0.1363.

Table 3 reports the 32-channel teacher’s performance as mean ± standard deviation across the 32 held-out participants. The teacher used the same LOSO splits and trial-level evaluation procedure described in Section 4.1. It serves as an empirical full-channel reference, not a guaranteed upper bound, because it did not outperform Proposed on every metric.

### 5.3. Confusion Matrix Analysis

**Figure 4:**
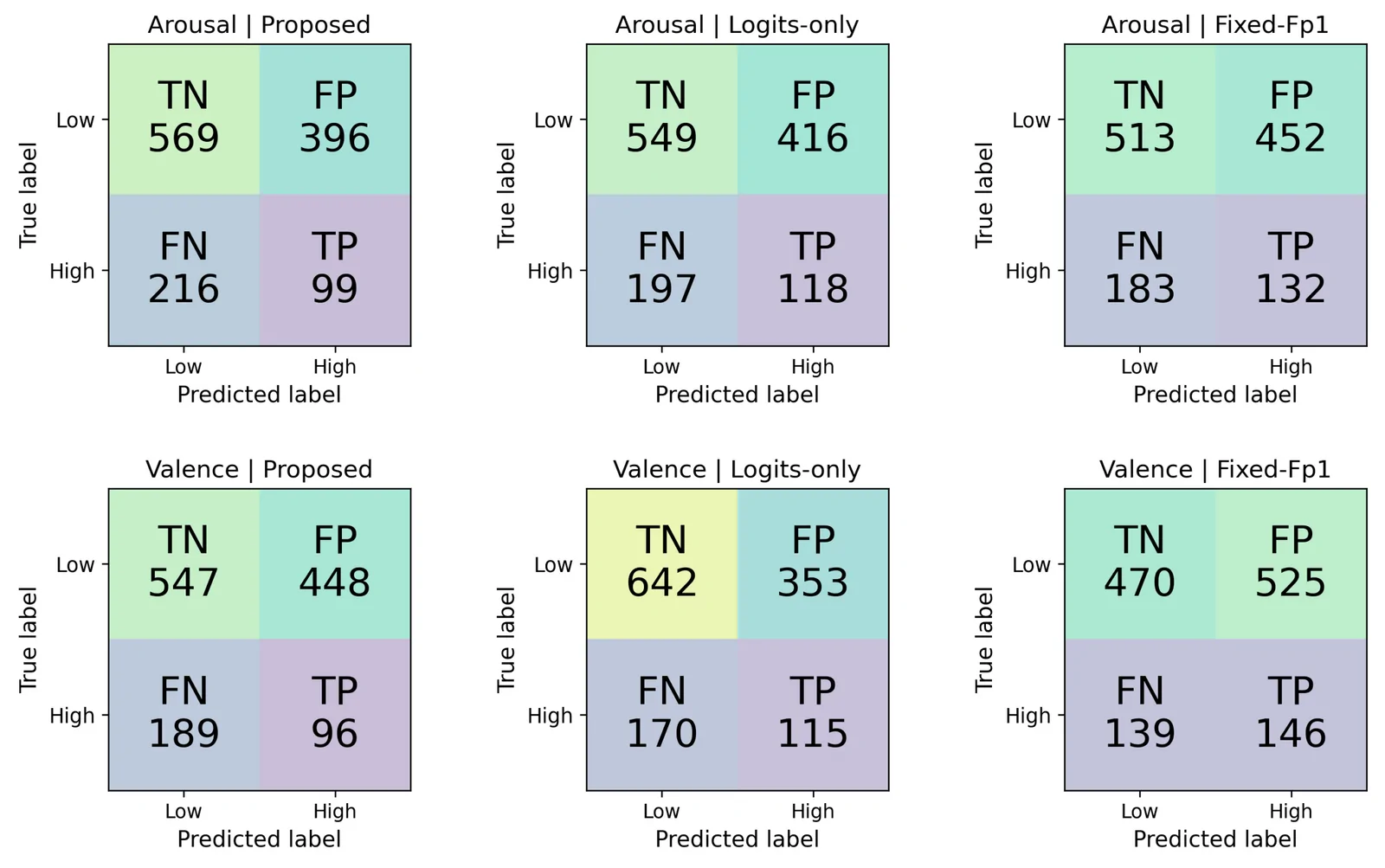
Pooled trial-level confusion matrices for Arousal (**top**) and Valence (**bottom**).

Figure 4 presents confusion matrices pooled across the 1280 held-out trials in each task. For Arousal, Proposed yielded TP = 99, FN = 216, TN = 569, and FP = 396. Logits-only yielded TP = 118, FN = 197, TN = 549, and FP = 416; Fixed-Fp1 yielded TP = 132, FN = 183, TN = 513, and FP = 452. Compared with either method, Proposed produced fewer false positives but more false negatives.

For Valence, Proposed yielded TP = 96, FN = 189, TN = 547, and FP = 448. Logits-only yielded TP = 115, FN = 170, TN = 642, and FP = 353; Fixed-Fp1 yielded TP = 146, FN = 139, TN = 470, and FP = 525. Compared with Fixed-Fp1, Proposed produced fewer false positives but more false negatives; compared with Logits-only, it produced more of both errors.

### 5.4. Prediction Bias Analysis

Positive-prediction rates were compared with the observed High-class prevalence for both tasks using the pooled held-out trial predictions. These rates describe prediction tendencies and are interpreted together with the confusion matrices.

For Arousal, 315 trials were labeled High (24.61%). Proposed generated 495 High predictions (38.67%), compared with 534 (41.72%) for Logits-only, 584 (45.63%) for Fixed-Fp1, and 436 (34.06%) for Channel-matched CE. All four methods predicted High more frequently than its observed prevalence. Proposed had a lower High-prediction rate than Logits-only and Fixed-Fp1, but a higher rate than Channel-matched CE.

For Valence, 285 trials were labeled High (22.27%). Proposed generated 544 High predictions (42.50%), compared with 468 (36.56%) for Logits-only, 671 (52.42%) for Fixed-Fp1, and 481 (37.58%) for Channel-matched CE. All four methods again predicted High more frequently than its observed prevalence. Proposed lay between Logits-only and Fixed-Fp1 and exceeded Channel-matched CE.

Together with Figure 4, these results show that the relationship between positive-prediction frequency and the balance of false positives and false negatives varies by task and comparator. A lower High-prediction rate should therefore be interpreted alongside both types of error rather than treated as an overall performance advantage.

### 5.5. Ablation Study

The ablation study compared six configurations on Arousal and Valence. Fixed-Fp1 uses supervised training on the predefined Fp1 channel. Learned channel only uses Sparsemax-based channel selection followed by retraining on the selected physical signal. Gate + logits KD adds logits distillation without frequency-band consistency, whereas Gate + band KD uses frequency-band consistency without logits distillation.

The w/o retraining configuration evaluates the validation-selected search model on its selected physical channel without further training. Proposed combines sparse gating, logits distillation, frequency-band consistency, and channel-locked retraining.

**Figure 5:**
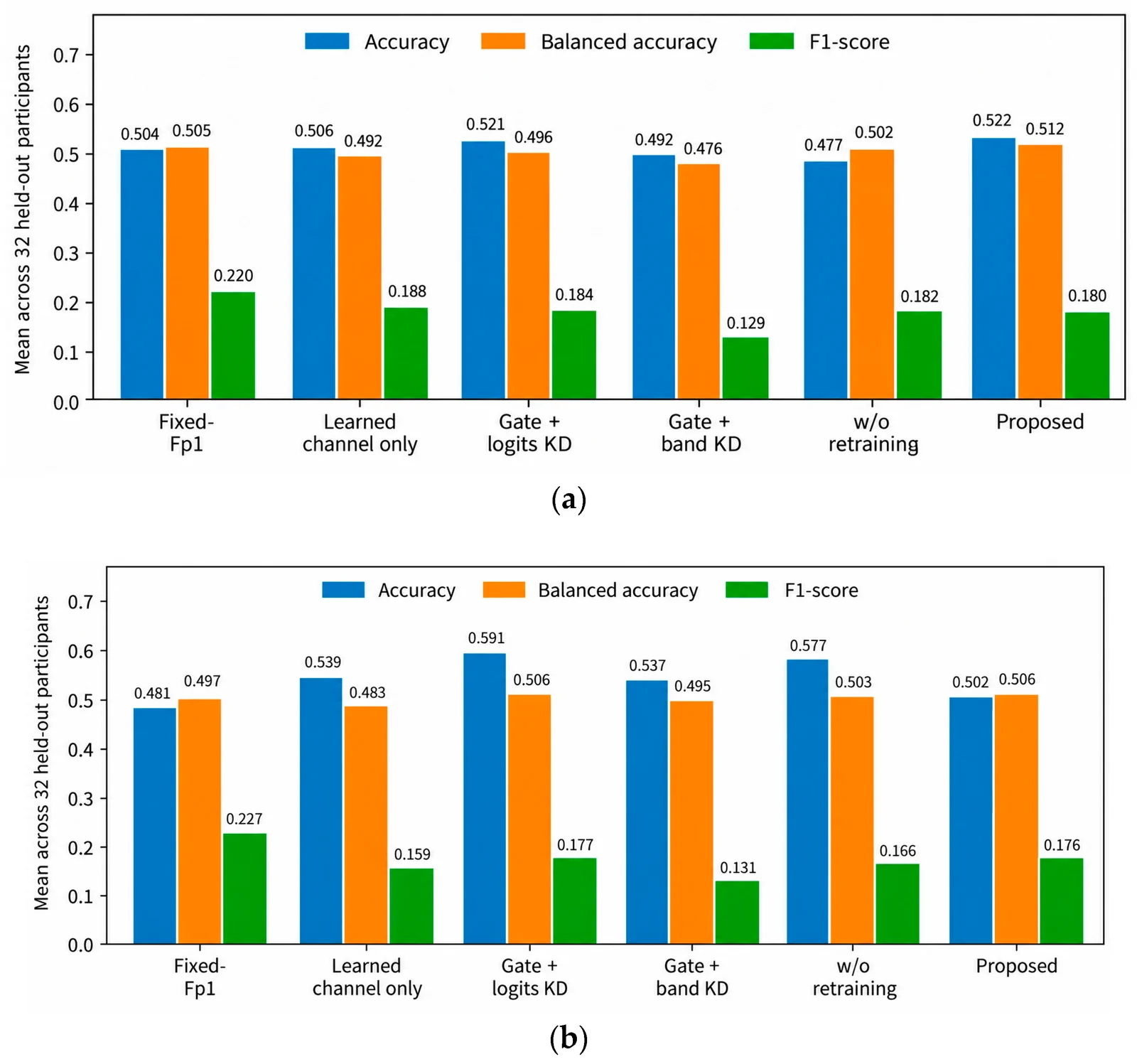
Ablation results for Arousal and Valence. (**a**) Arousal. (**b**) Valence.

**Figure 6:**
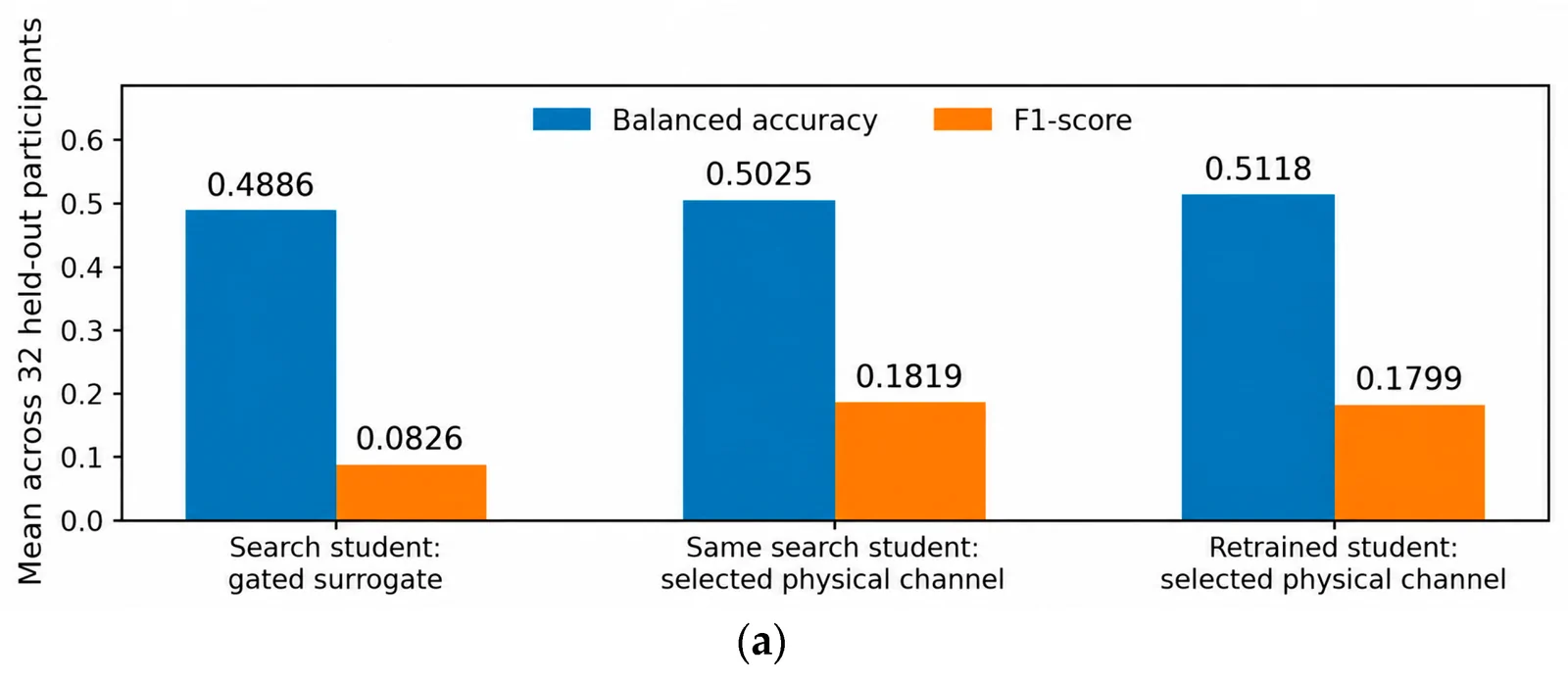

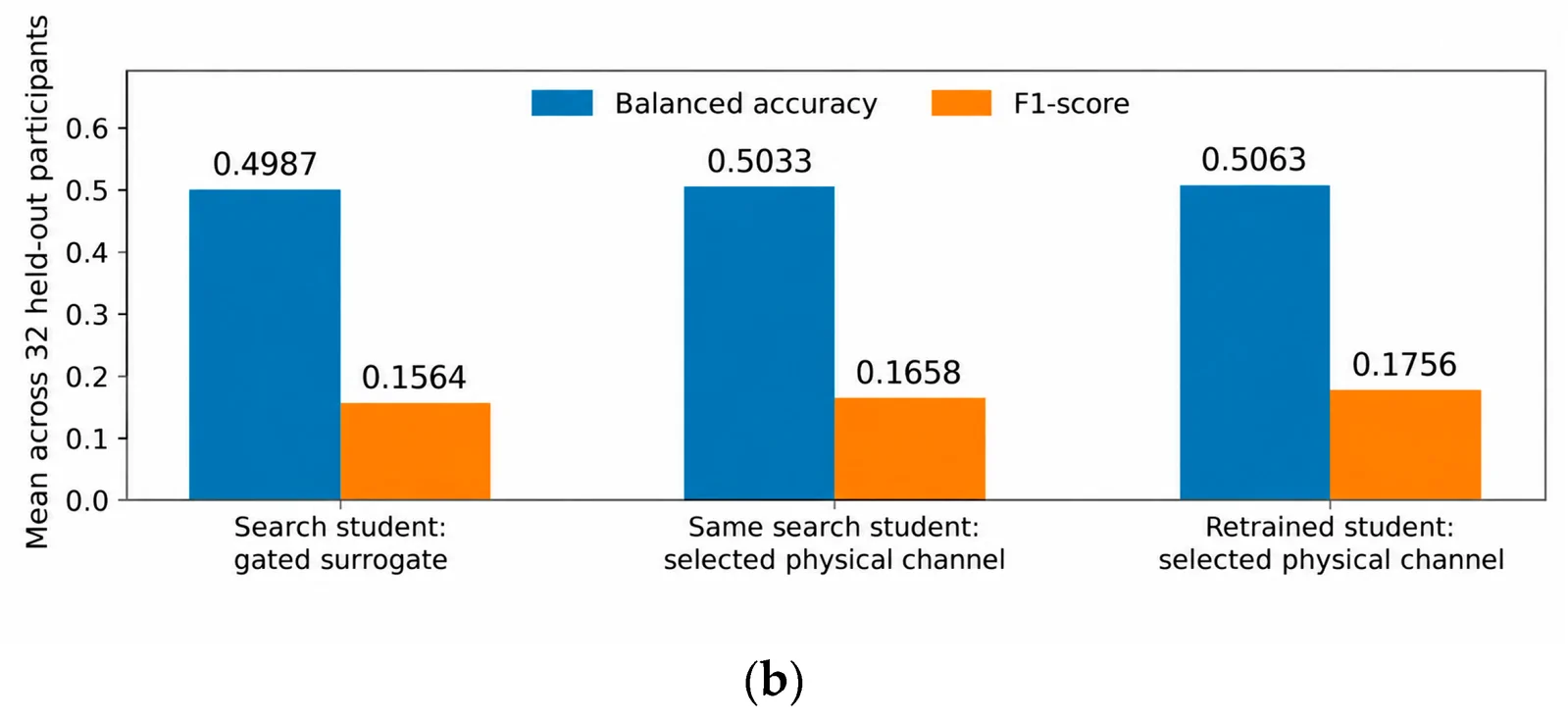
Input-configuration and retraining comparisons for Arousal and Valence. (**a**) Arousal. (**b**) Valence.

Figure 5 and Figure 6 summarize the ablation results. For Arousal, Proposed achieved a balanced accuracy of 0.5118 and an F1-score of 0.1799. The corresponding values were 0.4959 and 0.1838 for Gate + logits KD, and 0.4756 and 0.1285 for Gate + band KD. Compared with w/o retraining, accuracy increased from 0.4773 to 0.5219 and balanced accuracy from 0.5025 to 0.5118, whereas F1-score changed from 0.1819 to 0.1799.

For Valence, Proposed achieved a balanced accuracy of 0.5063 and an F1-score of 0.1756. The corresponding values were 0.5063 and 0.1770 for Gate + logits KD, and 0.4948 and 0.1315 for Gate + band KD. Compared with w/o retraining, accuracy decreased from 0.5773 to 0.5023, while balanced accuracy increased from 0.5033 to 0.5063 and F1-score from 0.1658 to 0.1756. These comparisons are descriptive and do not establish statistically significant independent gains from individual loss terms.

**Figure 7:**
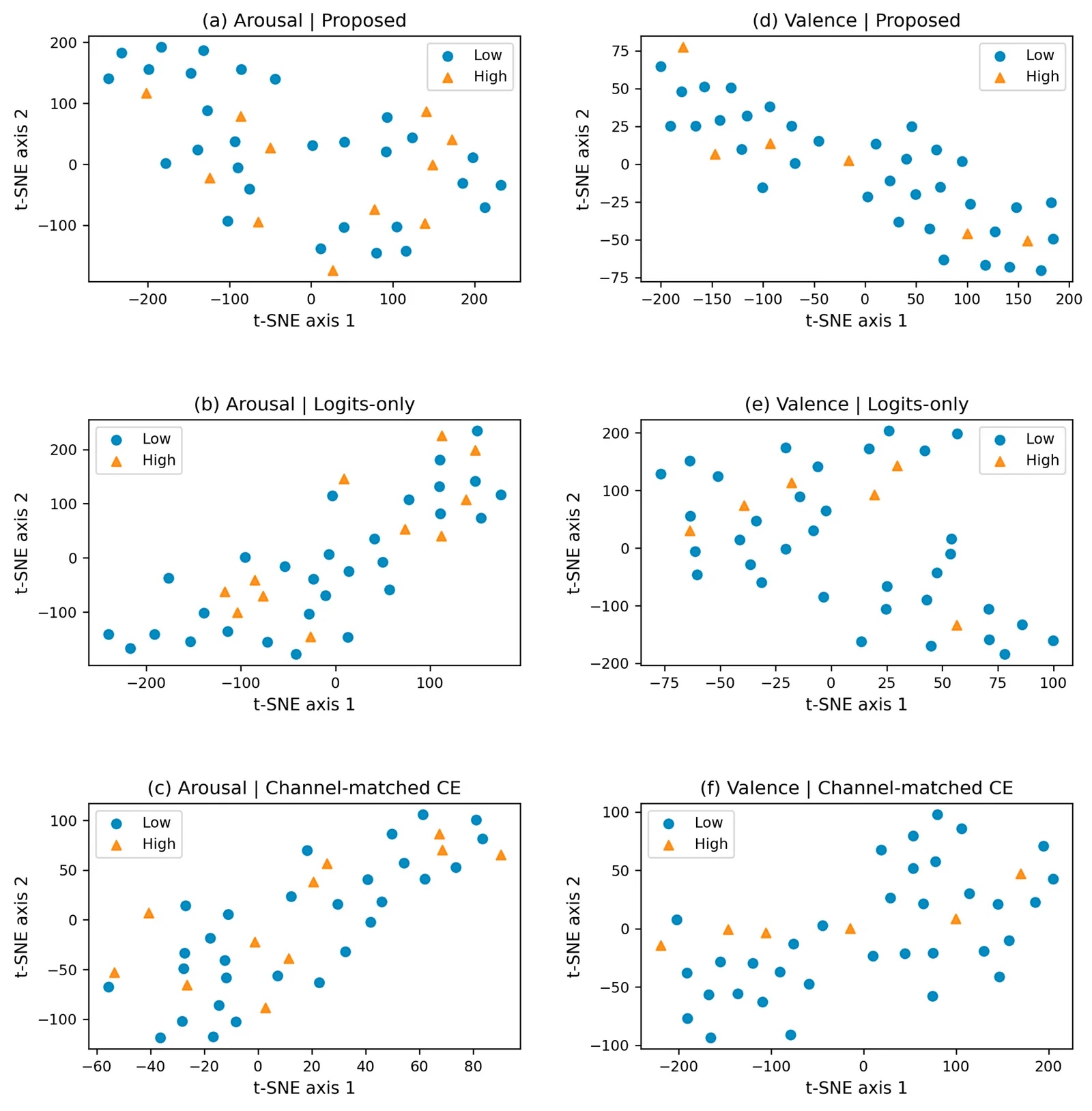
t-SNE visualizations of trial-averaged features from LOSO fold 01: Arousal (**left**) and Valence (**right**).

To examine the learned representations, t-SNE visualizations compare Proposed, Logits-only, and Channel-matched CE for both Arousal and Valence (Figure 7). These plots provide qualitative comparisons of feature distributions among different methods.

### 5.6. Spectral Analysis

**Figure 8:**
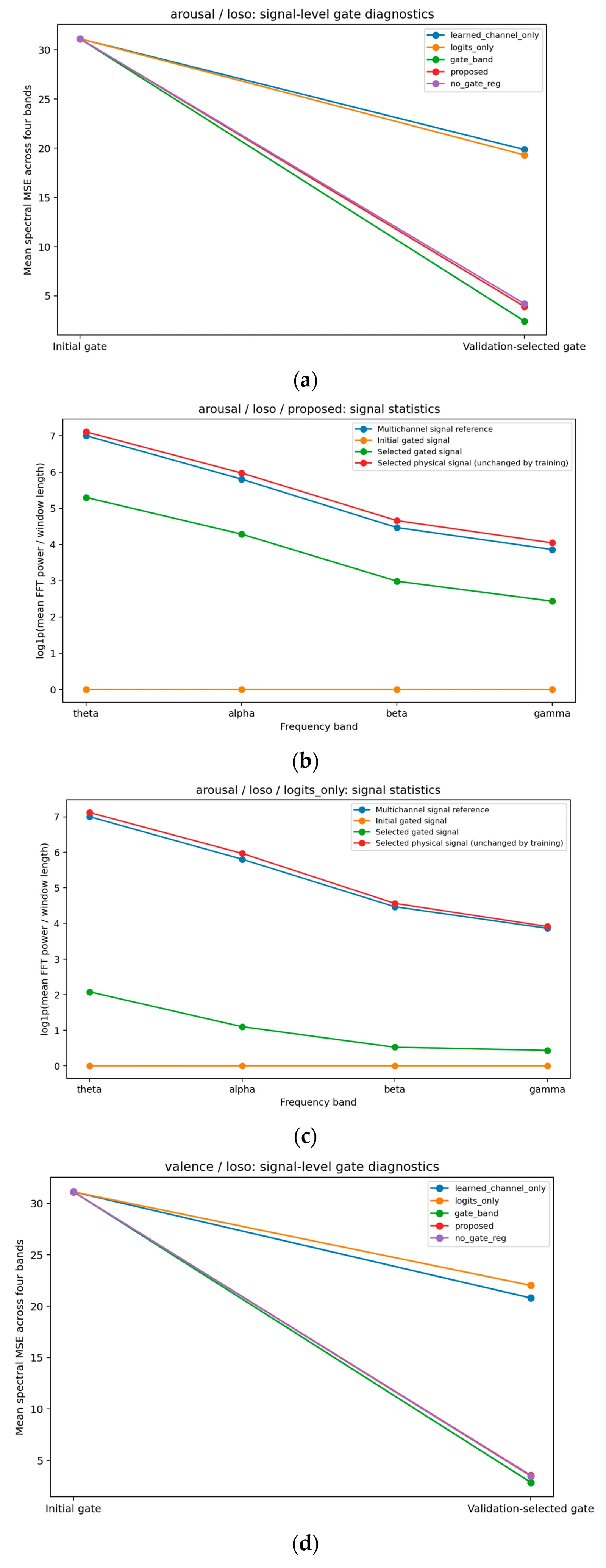

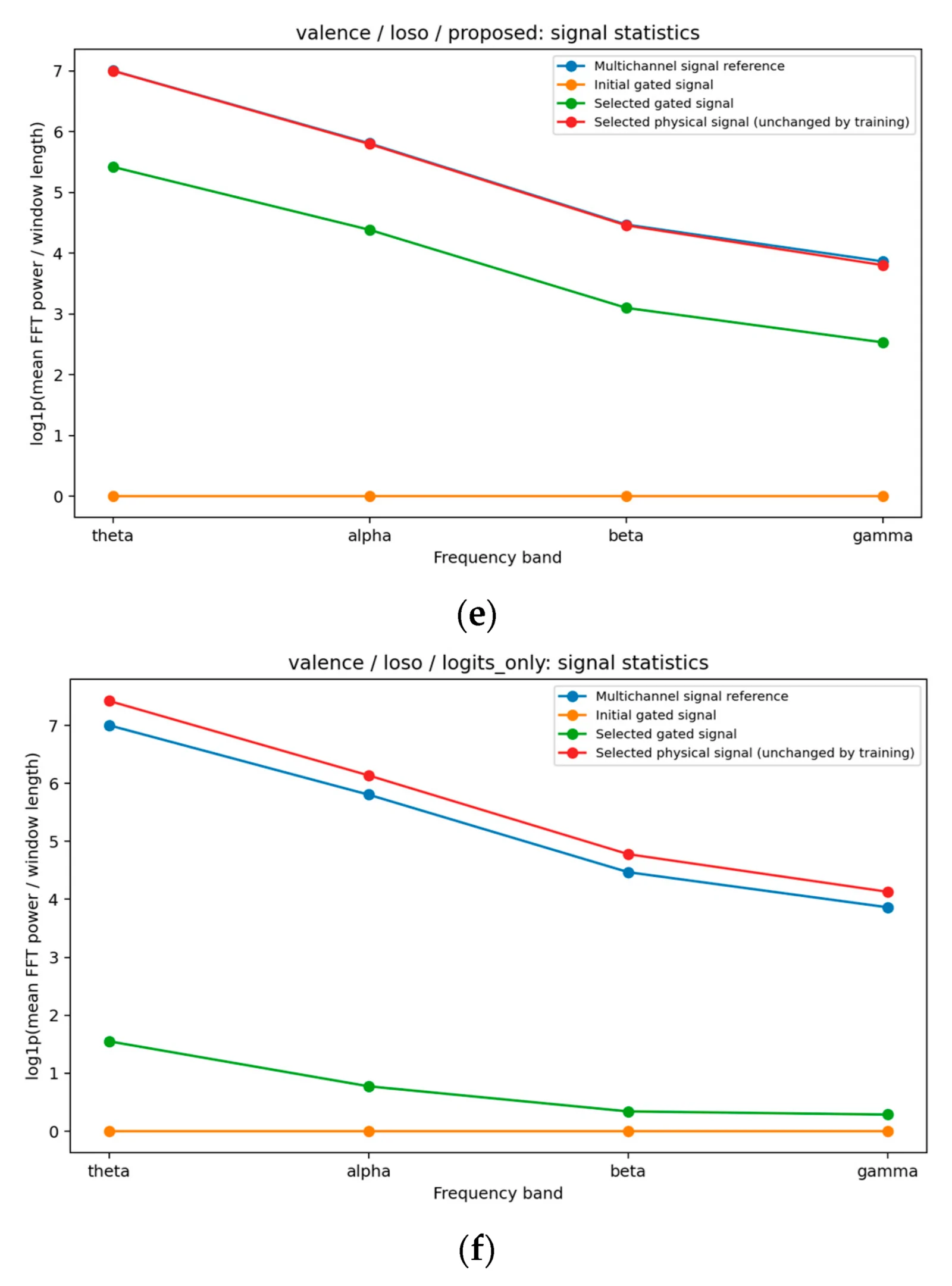
Signal-level spectral analysis for Arousal (**a**–**c**) and Valence (**d**–**f**). (**a**) Arousal frequency-band characteristics before and after gating. (**b**) Arousal spectral characteristics of Proposed. (**c**) Arousal spectral characteristics of Logits-only. (**d**) Valence frequency-band characteristics before and after gating. (**e**) Valence spectral characteristics of Proposed. (**f**) Valence spectral characteristics of Logits-only.

Frequency-band analysis was conducted for both Arousal and Valence using the θ, α, β, and γ bands (Figure 8). The plots compare the multi-channel signal reference with the initial gated surrogate, the validation-selected gated surrogate, and the selected physical input. They describe signal-level spectral characteristics during channel selection; the recorded physical signal is not changed by classifier training.

### 5.7. Sparsemax Channel Selection and Sparsity Analysis

**Figure 9:**
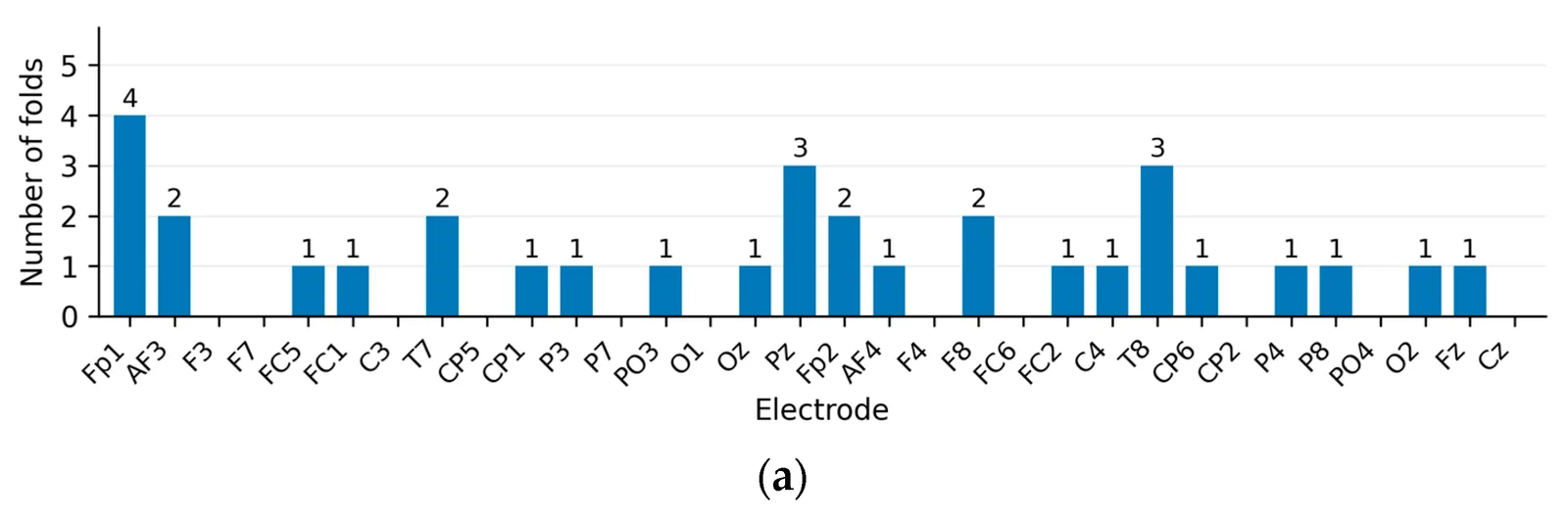

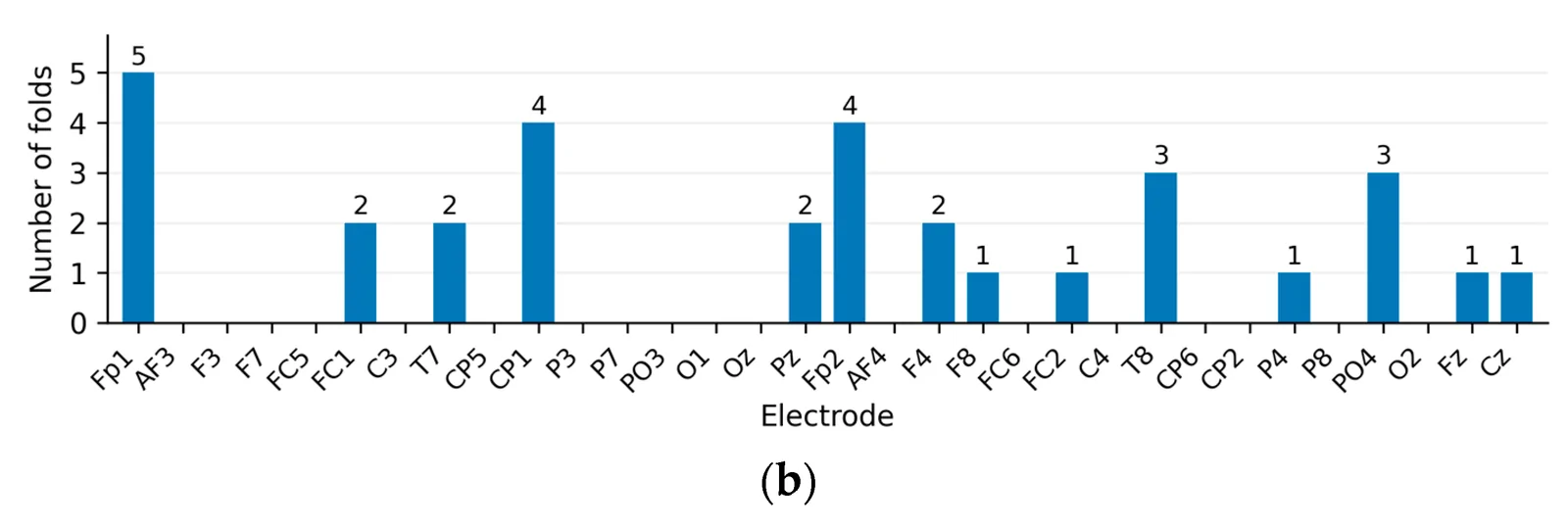
Electrode-selection frequencies of Proposed across 32 LOSO folds: (**a**) Arousal and (**b**) Valence. Each bar indicates the number of folds in which the corresponding electrode was selected as the final physical input channel. Each fold contributes one selected electrode per task.

Figure 9 summarizes the physical-channel selections across all 32 LOSO folds for Arousal and Valence. Proposed selected 21 distinct electrodes for Arousal and 14 for Valence. Fp1 was the most frequently selected electrode, appearing in 4 and 5 folds, respectively. These results show that the selected physical channel varied across folds rather than remaining identical throughout the evaluation.

The validation-selected gates retained 5–27 nonzero channel weights for Arousal and 5–29 for Valence. The final single-channel input was obtained by selecting the channel with the highest gating weight. Channel-matched CE used the channel selected by Proposed in the corresponding fold and task.

Table 4 reports frequency-band statistics for the physical channel selected by Proposed in each LOSO fold. For Arousal, the High–Low differences were 0.064, 0.010, −0.063, and −0.078 for θ, α, β, and γ, respectively. For Valence, the corresponding differences were −0.018, 0.035, −0.035, and −0.013. The absolute Cohen’s d values were below 0.05 in both tasks, indicating small descriptive differences in mean band power.

## 6. Discussion and Limitations

### 6.1. Discussion

Across both tasks, Proposed achieved higher subject-averaged balanced accuracy and F1-score than Channel-matched CE under matched electrode locations (Section 5.2). The observed differences therefore extend beyond selecting a particular physical channel. They support examining the proposed training strategy under the evaluated conditions, while the modest absolute performance limits broader conclusions.

A single-channel input restricts access to distributed spatial information and inter-channel relationships [8,9]. Multi-channel training does not restore access to unmeasured channels at inference. Moreover, teacher–student differences varied by task and metric, and the models used different training procedures. These comparisons therefore do not isolate the effect of channel reduction; the teacher remains an empirical reference rather than a strict upper bound.

Existing lightweight EEG approaches address related but distinct trade-offs. EEGNet [17] uses depthwise and separable convolutions to construct a compact EEG decoding architecture, emphasizing network compactness rather than specifying the single-physical-channel input constraint adopted here. A closer input-related comparison is the similarity-keeping distillation framework of Huang et al. [16], which transfers inter-sample similarity from a high-density teacher to support low-density motor-imagery decoding. Both frameworks use richer training information to mitigate reduced-channel input constraints. In the present framework, this aim is pursued through learnable single-channel selection, logits and frequency-band constraints, and retraining on the selected physical signal. These literature-based comparisons clarify design choices but do not replace direct benchmarking or establish a superior acquisition–performance trade-off over models not evaluated in this study.

The ablations in Section 5.5 did not show uniform gains from every component across tasks and metrics. Frequency-band consistency serves as an auxiliary signal-level constraint, not a sufficient source of classification supervision. Likewise, retraining adapts the student to the selected physical input but did not improve every metric. These findings distinguish the intended roles of the components from their observed performance effects.

The channel-selection results also indicate that the retained physical channel was not fixed across LOSO folds. Proposed selected 21 distinct electrodes for Arousal and 14 for Valence, with Fp1 being the most frequently selected electrode but appearing in only a minority of folds. Therefore, the present results support fold-dependent channel selection rather than a universal preference for a single electrode. This variability is consistent with the substantial inter-subject heterogeneity of EEG signals and motivates further stability analysis across random seeds and independent datasets.

### 6.2. Limitations

This study has several limitations. First, all experiments were conducted on the DEAP dataset. Although the revised evaluation includes both binary Arousal and Valence classification, these two tasks share the same recording protocol and participant population. The present findings therefore do not establish robustness across independent datasets. The evaluation also remains restricted to High/Low classification and does not establish applicability to multiclass emotion recognition or continuous emotion estimation. Future work should evaluate the framework on independent EEG emotion datasets using dataset-appropriate preprocessing and matched baselines.

Second, although the main evaluation uses 32-fold subject-independent LOSO, the component-level ablation analyses were not repeated across multiple random seeds with comprehensive paired statistical testing. Accordingly, the observed component effects should be interpreted descriptively, and stronger claims about the independent contribution or stability of individual loss terms require repeated experiments and statistical validation. The variability of the selected physical channel across folds also indicates that channel-selection stability should be examined further across seeds and datasets.

Third, the absolute classification performance remains modest and both tasks exhibit substantial class imbalance. The observed gains over Channel-matched CE in subject-averaged Balanced Accuracy and F1-score do not by themselves establish high absolute recognition performance. The purpose of the present comparisons is to evaluate training strategies under the same single-channel input constraint rather than to claim state-of-the-art emotion-recognition accuracy. Further improvement in minority-class recognition and overall robustness will be necessary before the framework can support practical emotion-monitoring applications.

Finally, the current experiments are based on offline DEAP recordings and do not establish performance on an actual wearable EEG system. Robustness to movement artifacts, electrode displacement, electrode–skin impedance changes, and environmental noise has not yet been evaluated. In addition, parameter counts, FLOPs, inference latency, memory consumption, and energy efficiency were not included in the quantitative evaluation, so computational-efficiency or real-time advantages are not demonstrated here. The present contribution should therefore be understood as a modeling approach that reduces the final signal-input requirement from 32 EEG channels to one selected physical channel, rather than as a fully validated wearable deployment solution. Future work will combine device integration, real-world robustness testing, computational profiling, and system-level cost assessment.

## 7. Conclusions

This study developed a multi-channel-to-single-channel EEG knowledge transfer framework for binary Arousal and Valence recognition. It combines Sparsemax-based channel selection, logits distillation, a signal-level frequency-band consistency objective, and retraining on the selected physical channel.

Under 32-fold LOSO evaluation, Proposed achieved accuracy, balanced accuracy, and F1-score values of 0.5219, 0.5118, and 0.1799 for Arousal, and 0.5023, 0.5063, and 0.1756 for Valence. Subject-averaged balanced accuracy and F1-score were higher than those of Channel-matched CE in both tasks, while performance differences and component effects remained task- and metric-dependent.

The framework provides a methodological basis for simplifying EEG acquisition, but the findings are limited to the evaluated DEAP conditions. Further evaluation is needed to establish statistical stability, cross-dataset applicability, computational efficiency, and performance on wearable EEG systems.

## Figures and Tables

**Table 1:** Trial-level label distributions for (**a**) Arousal and (**b**) Valence.

**(a) Arousal**			
**Dataset Split**	**Trials**	**Low Trials**	**High Trials**
Training	992	730–761	231–262
Validation	248	178–202	46–70
Test	40	21–39	1–19
Training + Validation	1240	926–944	296–314
Total	1280	965	315
**(b) Valence**			
**Dataset Split**	**Trials**	**Low Trials**	**High Trials**
Training	992	762–788	204–230
Validation	248	180–211	37–68
Test	40	19–38	2–21
Training + Validation	1240	957–976	264–283
Total	1280	995	285

**Table 2:** Descriptive statistics of all-channel mean frequency-band log power for (**a**) Arousal and (**b**) Valence.

**(a) Arousal**						
**Band**	**Low n**	**High n**	**Low Mean Log Power**	**High Mean Log Power**	**High − Low**	**cohen_d**
alpha	995	315	5.788	5.858	0.070	0.065
beta	995	315	4.450	4.523	0.073	0.070
gamma	995	315	3.842	3.921	0.079	0.062
theta	995	315	6.970	7.101	0.131	0.092
**(b) Valence**						
**Band**	**Low n**	**High n**	**Low Mean Log Power**	**High Mean Log Power**	**High − Low**	**cohen_d**
alpha	995	285	5.805	5.808	0.003	0.003
beta	995	285	4.476	4.440	−0.037	−0.035
gamma	995	285	3.875	3.816	−0.059	−0.046
theta	995	285	7.032	6.900	−0.132	−0.094

**Table 3:** Trial-level performance of the 32-channel teacher.

Metric	Arousal	Valence
Accuracy	0.6102 ± 0.2150	0.5469 ± 0.2626
Balanced Accuracy	0.5063 ± 0.0525	0.5133 ± 0.0414
F1-score	0.1215 ± 0.1763	0.1936 ± 0.1946
Precision	0.0982 ± 0.1474	0.1578 ± 0.2116
Recall/TPR	0.2713 ± 0.4100	0.4505 ± 0.4501
TNR	0.7413 ± 0.3644	0.5762 ± 0.4379

**Table 4:** Frequency-band log-power statistics for the selected physical channels.

**(a) Arousal**						
**Band**	**Low n**	**High n**	**Low Mean** **Log Power**	**High Mean** **Log Power**	**High − Low**	**Cohen’s d**
theta	965	315	7.093	7.157	0.064	0.033
alpha	965	315	5.973	5.983	0.010	0.007
beta	965	315	4.676	4.614	−0.063	−0.041
gamma	965	315	4.065	3.987	−0.078	−0.043
**(b) Valence**						
**Band**	**Low n**	**High n**	**Low Mean** **Log Power**	**High Mean** **Log Power**	**High − Low**	**Cohen’s d**
theta	965	285	7.004	6.985	−0.018	−0.011
alpha	965	285	5.787	5.822	0.035	0.026
beta	965	285	4.463	4.428	−0.035	−0.026
gamma	965	285	3.804	3.791	−0.013	−0.008

Note: n denotes trials; High − Low is the difference in mean log power.

## Data Availability

A publicly available dataset was analyzed in this study. The DEAP dataset can be accessed at https://www.kaggle.com/datasets/manh123df/deap-dataset (accessed on 26 July 2026).
